# Validated HPLC method for simultaneous quantitative determination of dimethylcurcumin and resveratrol in pluronic-F127 nanomicelles: Formulation with enhanced anticancer efficacy

**DOI:** 10.1016/j.mex.2023.102457

**Published:** 2023-10-20

**Authors:** Wasiporn Arakkunakorn, Watchara Pholthien, Warayuth Sajomsang, Abdul Basit, Sasikarn Sripetthong, Sirinporn Nalinbenjapun, Chitchamai Ovatlarnporn

**Affiliations:** a Drug Delivery System Excellence Center, Prince of Songkla University, Hat Yai, Songkhla 90112, Thailand; b Department of Pharmaceutical Chemistry, Faculty of Pharmaceutical Sciences, Prince of Songkla University, Hat Yai, Songkhla 90112, Thailand; c National Nanotechnology Center (NANOTEC), National Science and Technology Development Agency (NSTDA), Thailand Science Park, Pathum Thani 12120, Thailand

**Keywords:** *HPLC*, Quantitative determination, HPLC, Dimethylcurcumin, Resveratrol, Pluronic F-127 micelles, Anti-cancer

## Abstract

Nano-micelles offer a promising vehicle for the delivery various therapeutically significant biologicals. Development of convenient and efficient chromatographic methods for the quantitative determination of the active pharmaceutical ingredients in such systems is of immense importance. In this study pluronic-F-127 nano-micelles were prepared and loaded with dimethylcurcumin (DMC) and resveratrol (Res). A simple, convenient and effective HPLC method was developed for the quantitative estimation of DMC and Res in the polymeric nano-micelles through a single injection. A reverse-phase ACE® C18 column (250 mm × 4.6 mm) was used with a gradient mobile phase system consisting of 1 % MeOH and 0.1 % H_3_PO_4_:100 % acetonitrile at 1 mL/min flow rate with UV detection for Res, and fluorescence detector for DMC. The calibration curves generated for both the compounds were found linear with r^2^ values of 1.000 over a concentration range of 2–25 µg/mL with low limit of detection (LOD) values of 0.37 and 0.16 µg/mL for DMC and Res respectively and limit of quantification (LOQ) values of 1.23 and 0.55 µg/mL for DMC and Res respectively. Similarly, accuracy was found in a range of 98.80 -102.47 % for DMC and 100.58–101.77 % for Res. Furthermore, the within-run precisions (%RSD) were 0.073 - 0.444% for DMC and 0.159 - 0.917% for Res, while between-run precisions (%RSD) were 0.344 - 1.47 for DMC and 0.458 - 1.651 for Res. Moreover, the DMC with Res co-loaded nanomicelles showed higher activity against MCF-7 and MDA-MB 231 compared to DMC and Res alone. Overall, this study presented a simple, convenient, precise and accurate method for the quantitative determination of DMC and Res in polymeric nano-micelles which have anticancer potential.

• A simple HPLC for the quantitative determination of DMC and Res in nanomicelles having anti-cancer potential.
• Non complicate with high degree of recoveries of sample preparation process.
• This method can be used to determine a mixture of DMC and Res in pharmaceutical formulation in single injection.

## Graphical abstract

**Figure:**
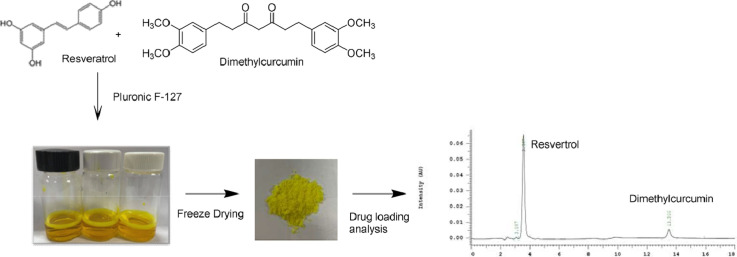

Specifications table

**Table:** 

Subject area:	Pharmacology, Toxicology and Pharmaceutical Science
More specific subject area:	*Method validation*
Name of your method:	*HPLC*
Name and reference of original method:	*None*
Resource availability:	*None*

## Background

**Fig. 1:**
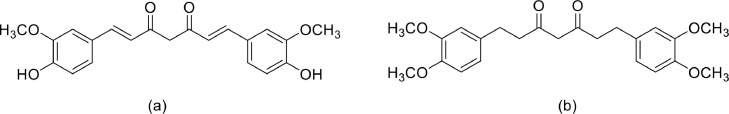
Structures of curcumin (a) and dimethylcurcumin (b).

Curcumin (Fig. 1a) is a bis-*R*-unsaturated diketone, which is a hydrophobic polyphenol derived from the rhizome of turmeric (*Curcuma longa*). It is known to have chemo-chemopreventive [30] and anticancer activities [32,34]. Dimethylcurcumin (DMC, (1*E*, 6*E*) −1, 7-bis (3, 4-dimethoxyphenyl) hepta-1, 6-diene-3, 5‑dione) is a synthetic curcumin analog (Fig. 1b) structurally related to the structure of curcumin. DMC was reported to exhibit anti-breast, anti-prostate, and anti-leukemia cancers, anti-trypanosomal and anti-leishmanial, anti-oxidation, and anti-mycobacterial activities [19,^21^,24]. Furthermore, DMC showed higher activity in anti-cancer, antioxidant, and anti-mycobacterial pathways than curcumin [11,19].

**Fig. 2:**
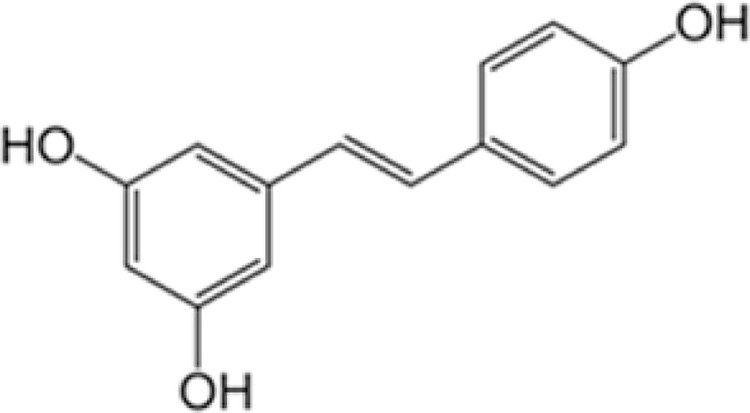
Structure of trans-resveratrol.

Resveratrol (Res, Fig. 2) is a naturally occurring polyphenol derived from many sources of natural products [10,31]. The curcumin has been explored for its valuable pharmacological activities, such as cardioprotective, neuroprotective, and chemopreventive, along with anti-aging properties [6,^16^,27]. However, at the moment, the clinical use of these compounds is not authorized anywhere. Res is known to have a chemo-preventive effect [7,14] *via* several pathways, including inhibition of COX-1 and −2, suppression of protein kinase-C, and AP-1-mediated gene expression. Suppression of NFκB by Res by decreasing the phosphorylation and degradation of IκBα was reported. Moreover, Res induces cancer cells apoptosis via a p53-dependent pathway involving mitogen-activated protein kinase **(**MAPK), extracellular signal-regulated kinases (ERKs), c-Jun N-terminal **kinase (**JNKs), or p38 kinase activities [15].

However, using DMC and Res in effective treatment is limited by their pharmacokinetic properties, since they have poor aqueous solubility [8,^13^,20]. Moreover, the metabolism of Res is extremely rapid and extensive, resulting in a short plasma half-life [36]. It is therefore no surprise that a number of studies have focused on the preparation of nanomicelles of these compounds in order to improve their solubilities [4]. Several types of polymers were investigated for solubility improvement of Res, including Pluronic [1,^2^,^12^,^17^,^25^,33]. In 2015, Liu and co-workers utilized *N*-*t*-butoxycarbonyl-phenylalanine terminated mono-methoxyl poly (ethylene glycol)-*b*-poly (ε-caprolactone), or mPEG-PCL-Phe (Boc) to develop a polymeric micellar formulation of DMC by thin film hydration method and found that it improve pharmacokinetic, biodistribution, as well as tumor targeting properties [22]. The amphiphilic deblock-copolymer of monomethoxy poly(-ethylene glycol) (mPEG)-poly (lactic acid) (PLA), mPEG-PLA, a FDA approved polymer, was used as a drug carrier to encapsulate the anti-cancer agent doxorubicin (DOX) with the potent antioxidant DMC, resulting in anti-cancer potency in improved and reduced toxicity of DOX [28]. The combination of curcumin and its analogs with other bioactive compounds, including Res, was found to increase anti-cancer, anti-malarial, and anti-inflammatory activities [18]. Carlson and team prepared Res and curcumin co-loaded in Pluronic® micelles and co-administered with DOX *in vitro* found that the system provided cardioprotective effects while maintaining and improving anti-ovarian cancer *in vitro* [9]. We recently reported for the first time Pluronic F-127 nanomicelles containing DMC and Res (in the ratio of 1:5) for solubility improvement prepared by the thin film hydration method [5]. In this article, another method was applied to construct the nano-micelles containing DMC and Res (in the ratio of 1:5) by solvent casting and equilibrium methods according to the procedure reported [29]. To obtain a quantitative determination of both DMC and Res in the obtained nano-micelles, HPLC was selected. However, no report was published to determine these types of compounds together in one injection of HPLC sample. We herein report a simple and effective reverse-phase HPLC method for the determination of both DMC and Res in the obtained Pluronic F-127 nanomicelles in one injection. The method was well validated and can be applied to the quantitative determination of DMC and Res in the obtained Pluronic F-127 nanomicelles sample as well as their solubility study.

## Method details

### Chemical and reagents

Resveratrol was purchased from Carbosynth Limited, Compton, Berkshire, UK. Pluronic F-127 was purchased from Sigma-Aldrich, Germany. Analytical grade acetonitrile was obtained from Fluka, Germany. DMC was synthesized in our laboratory according to our method [24] with a purity of over 99 %. A nylon-membrane syringe filter with pore sizes of 0.22 µm was purchased from Vertical Chromatography, Thailand. Analytical grade dimethyl sulfoxide (DMSO) was purchased from Labscan, Thailand. All other reagents were of analytical grade and used without further purification.

### Preparation of pluronic nanomicelles containing DMC, Res, and co-loaded DMC-Res as QC samples

The micelles were prepared in accordance with the protocols of the simple equilibrium method as previously described with little modification [1]. Briefly, in this method, 5 mL of 100 mg/mL Pluronic- F127 in deionized water was mixed with excess amounts of all compounds [100 mg of Res and 20 mg of DMC]. The mixtures were stirred at 200 rpm at room temperature, protected from light for 3 days to allow equilibrium to be achieved. After that, the samples were centrifuged at 4000 rpm for 5 min, and the supernatant was filtered using a (0.2 µm nylon filter), resulting in clear solutions. The obtained nanomicelles were subjected to freeze drying, providing solid samples, and they were used in the assay by RP-HPLC. All samples were prepared in triplicate.

### entrapment efficiencies (%EE) and% loading capacities (%LC) determination

All dried samples of Pluronic nanomicelles containing DMC and Res were taken and diluted with acetonitrile to give final solutions having concentrations of about 12 µg/mL for DMC and 12.5 µg/mL for Res. They were filtered through a nylon syringe filter (pore size 0.22 mm) and subjected to be analyzed by the HPLC system. Concentrations of DMC and Res in samples were calculated from the calibration curves of the standard solutions.% Encapsulation efficiencies (%EE) and% loading (LC) of DMC and Res were calculated using the bellowed Eqs. (1) and (2), respectively

(1) $$\%EE=\;\left(\frac{\text{wt}\;\text{of}\;\text{each}\;\text{drug}\;\text{entrapped}\;\text{in}\;\text{the}\;\text{micelles}\;\text{samples}\;}{\text{wt}\;\text{of}\;\text{drugs}\;\text{added}\;\text{in}\;\text{micelles}\;\text{preparation}}\right)\times \;100$$

(2) $$\%LC=\;\left(\frac{\text{wt}\;\text{of}\;\text{each}\;\text{drug}\;\text{entrapped}\;\text{in}\;\text{the}\;\text{micelles}\;\text{samples}\;}{\text{wt}\;\text{of}\;\text{total}\;\text{amount}\;\text{of}\;\text{micelles}}\right)\times \;100$$

### Analytical protocol

HPLC analysis was carried out using a Hitachi CM-5000 equipped with a Hitachi Chromaster 5430 diode array detector and a Chromaster 5440 fluorescence detector with an autosampler. Data analysis was performed using a Chromaster software. The samples were separated on a reverse phase HPLC (ACE^Ⓡ^, C-18, 5 mm, 250 mm x 4.6 mm). A mobile phase gradient system of A, a mixture of 1 % MeOH + 0.1 % H_3_PO_4_ (pH 2.0), and B, acetonitrile was used during analysis with 18 mins run time. The% ratios of mobile phases A / B were 55/45 (0–7 mins), 30/70 (7.01–16 mins), and 55/45 (16.01–18 mins). The flow rate was maintained at 1 mL/min and at 40 °C column oven setting. The autosampler was set to inject standards and samples at 5 µl. Res was detected by a photodiode array detector at 306 nm, whereas DMC was determined by a fluorescence detector with an excitation wavelength of 420 nm and an emission wavelength of 470 nm. The two detectors were serially set for the detection of the compounds. The eluent flow from the column was made first through UV detector and then through the florescence detector. The flow from the UV detector reached the fluorescence detector in a few seconds.

#### Preparation of standard solutions

Stock solutions (1 mg/mL) of DMC and Res were prepared by separately dissolving 10 mg of DMC and 10 mg of Res in DMSO in 10 mL volumetric flasks. The stock solutions were then serially diluted with acetonitrile, providing 5 different concentrations of standard solutions at 2, 4, 8, 10, 15, and 25 µg/mL, respectively. They were analyzed by the HPLC system, and the results were used for calibration curves construction.

#### Mobile phase compositions

Mobile phases A and B in HPLC gradient system, were prepared as follows. Mobile phase A was a mixture of 1 % MeOH and 0.1 % H_3_PO_4_ with a pH adjusted to 2.0 ± 0.1 and mobile phase B was 100 % acetonitrile. Each mobile phase was kept in a separate bottle and freshly prepared by filtration through a 0.45 mm membrane filter and sonication for 10 mins before use.

### Analytical method validation

Validation parameters of the analytical method for DMC and Res entrapped in pluronic-F127 micelles were optimized, including linearity, range, precision, selectivity, accuracy, limit of detection (LOD), and limit of quantitation (LOQ). The optimized method was validated according to ICH guidelines for the validation of analytical methods [35].

#### Selectivity

Selectivity is the ability of an analytical method to differentiate and quantify the analyte in the presence of other components in the sample. The specificity and selectivity of DMC and Res samples were analyzed by comparing the samples with blank pluronic-F127 micelles. The UV–visible absorbance of Res was recorded at 306 nm. The fluorescence emission of DMC was recorded using an excitation wavelength of 420 nm and an emission wavelength of 470 nm.

#### Linearity and range

Standard solutions of DMC and Res at different concentrations covering the range of 2–25 µg/mL were utilized in the calibration curves construction. The analysis was performed repeatedly for at least 5 determinations per concentration. The peak area was plotted subsequently against the concentration of each reference standard solution. The linearity was assessed using linear regression using the excel program. The curves were considered linear if the coefficient of correlation (r^2^) was equal to or greater than 0.99 [3].

#### Accuracy

The accuracy of the current analytical method was demonstrated by recovery studies. Three concentrations (low, medium, and high) of working standard solutions of both compounds were prepared having concentration of 8, 10, and 12 µg/mL. They were analyzed according to the developed HPLC conditions in triplicate. The recoveries were calculated by comparison with the calibration curves. The recovery (%) was determined using the Eq. (3) below.

(3) $$Recovery\;\left(\%\right)=\;\frac{C_{obs}}{C_{spiked}}\times 100$$

Which C_obs_ refers to recovered concentration of each standard

S_piked_ refers to spiked concentration of standard solution of each standard

#### Repeatability

The repeatability of an analytical method is the similarity of individual measurements of an analyte when the procedure is repeated repeatedly. Precision was evaluated based on the relative standard deviation (%RSD) of the repeated analyses.

#### Inter-day precision

Inter-day precision analysis was determined by an analysis of the QC samples with concentrations of 2, 4, 8, 10, 15, and 25 µg/mL. Each QC sample was determined three times repeatedly in the same day at different periods of time. The repeatability of the analytical method was evaluated from RSD (%) results.

#### Intra-day precision

Intra-day precision analysis studies were determined by the analysis of QC samples with concentrations of 2, 4, 8, 10, 15, and 25 µg/mL in three different days. The repeatability of the analytical method was evaluated using RSD (%) results.

#### Limit of detection (LOD) and limit of quantitation (LOQ)

The detection limit of an analytical method is the lowest amount of the analyte that can be detected. LOD value indicates the sensitivity of the analytical method. The quantitation limit of the analytical method is the lowest amount of the analyte that can be quantitatively determined with suitable precision and accuracy. In this study, LOD and LOQ were determined using standard solutions of DMC and Res and compared with the results obtained from blank nanomicelles. The signal to noise (S/N) ratio of each standard signal was calculated. LOD and LOQ were calculated from the calibration curves of DMC and Res using the following Eqs. (4) and (5):

(4) $$\text{LOD}=\frac{3.3\sigma }{S}$$

(5) $$\text{LOQ}=\frac{10\sigma }{S}$$

σ is the standard deviation of the response and S is the slope of the calibration curve.

#### Cytotoxicity evaluation using MTT assay

Breast cancer cell line (MCF-7) and triple-negative breast cancer (MDA-MB-231) cell lines were maintained in Dulbecco's Modified Eagle Medium (DMEM), which has low and high glucose with 10 % fetal bovine serum, respectively [23]. The cells were maintained at 37 °C under 5 % carbon dioxide and 90 % relative humidity. The cytotoxicity activity of different test samples was determined by using a standard 3-[4,5-*dimethylthiazol*-2-*yl*]−2,5-diphenyl tetrazolium bromide (MTT)-based colorimetric assay at 550 nm. Samples including blank medium (control) or medium that contain samples containing DMC, Res, and DMC-Res co-loaded micelles were subjected to testing. The percentage of cell viability was then calculated using the following equation. The cytotoxicity activity of test samples was expressed in IC_50_ values calculated from Eq. (6).

(6) $$\text{Cytotoxicity}\;\left(\%\right)=\left(\frac{\left(Ab_{550}\text{control}\;-\;Ab_{550}\text{sample}\right)}{Ab_{550}\text{control}}\;\right)\times 100$$

#### Statistical analysis

A one-way ANOVA was used to compare the results of all the studies. When *p* < 0.05 is present, the differences were considered statistically significant. Statistical Package for the Social Sciences (SPSS) Version 22.0 software was used for the statistical analysis.

## Discussion and conclusion

### Preparation of nanomicelles and loading of dimethylcurcumin (DMC) and resveratrol (Res)

The development of resistance and barriers in the way of anticancer agents to reach the site of action in the target cells makes them less efficacious. This further leads to poor compliance, which limits their applications. In recent times, the combination therapies have sorted out the issue of physicochemical barriers. Loading of the combination of therapeutic moieties in polymeric nano-micelles has been observed with a marked increase in their anticancer effects [26]. Low water solubility of resveratrol is one of the key problems, limiting the uptake from foods or in pharmaceutical form [13]. It is therefore still focused on improving its water-dispersibility in order to increase its chemical stability and bioavailability [40, 41]. There is an intense need for developing and validating methods for quantitative estimation of the active ingredients in such carrier systems. In this report, we prepared nanomicelles loaded with dimethylcurcumin (DMC) and resveratrol (Res) and developed a method for their quantitative determination in the nanomicelles.

### The entrapment efficiency (EE) of DMC-resveratrol loaded pluronic F127 micelles

Table 1 shows the analysis results for the%entrapment of DMC and Res retained in DMC, Res-loaded pluronic F127 micelles. The%entrapment of DMC in the DMC-Pluronic micelles was 8.13 ± 0.12 %, and the%entrapment of Res in the Res-Pluronic micelles was 48.20 ± 0.52. Upon retention of DMC and Res together, DMC-Res-loaded pluronic F127 showed an increase in%entrapment with DMC = 23.18 ± 0.51 (about 3 folds) and Res = 53.43 ± 0.30 (about 1.1 folds).

**Table 1:** %Entrapment efficiencies of DMC, Res pluronic micelles.

Sample	%Entrapment efficiency	
	DMC	resveratrol
DMC-Pluronic micelles	8.13±0.12	–
Res-Pluronic micelles	–	48.20±0.52
DMC-Res-Pluronic micelles	23.18±0.51	53.43±0.30

All the values are represented as mean ± SD., (*n* = 3).

### Solubility of DMC, Res, and DMC-Res loaded pluronic F127 micelles

**Fig. 3:**
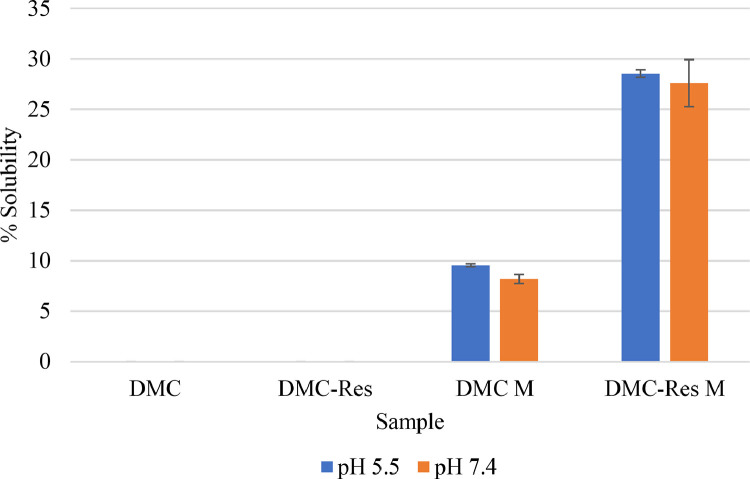
Solubility of pure DMC (DMC), DMC-Pluronic F127 micelles (DMC-M) and DMC.Res-Pluronic F127 micelles (DMC-res-M) at pH=5.5 and pH=7.4.

**Fig. 4:**
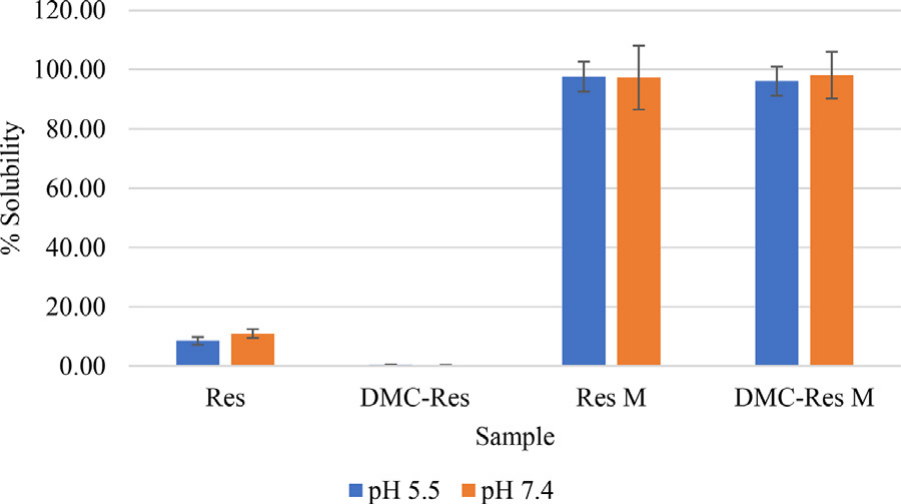
Solubility of pure Res, Res-Pluronic F127 micelles and DMC.Res-Pluronic F127 micelles at pH=5.5.

Fig. 3, Fig. 4 show the results obtained from the solubility study of the entrapped DMC and Res in DMC-Res loaded pluronic F127 micelles compared with the pure drug in buffer solutions of pHs 5.5 and 7.4, respectively. It was found that pure DMC and Res had very low solubility in both pHs, even though they were in a mixture of an equal amount of each. DMC in DMC loaded pluronic micelles cloud improve its water solution about 10 times in both pHs. The significant improvement of water solubilities properties in both pHs of DMC about 3 times in DMC loaded pluronic micelles and 30 times when co loaded with Res, respectively higher than pure DMC was observed (Fig. 3). Similar results were found with Res (Fig. 4). Pure Res and Res in a mixture with DMC has very low water solubility in both pHs. But when Res retained pluronic micelles, either in single loaded or co loaded with DMC, complete solubility property in both pHs was observed.

### Development and validation of HPLC method for the determination of DMC and Res in nanomicelles

**Fig. 5:**
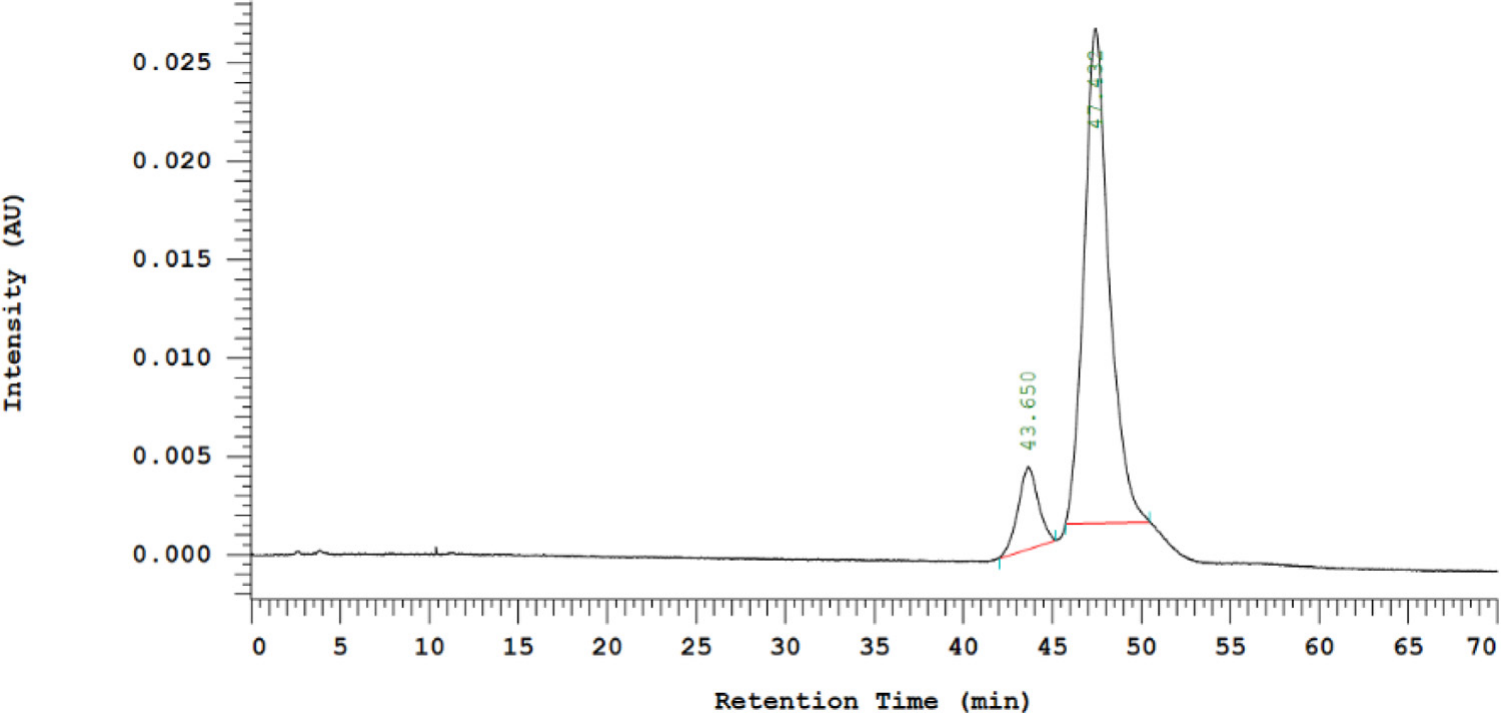
HPLC chromatogram of 10 mg/ml DMC solution under fluorescence detector (excitation wavelength 420 nm; emission wavelength 470 nm).

**Fig. 6:**
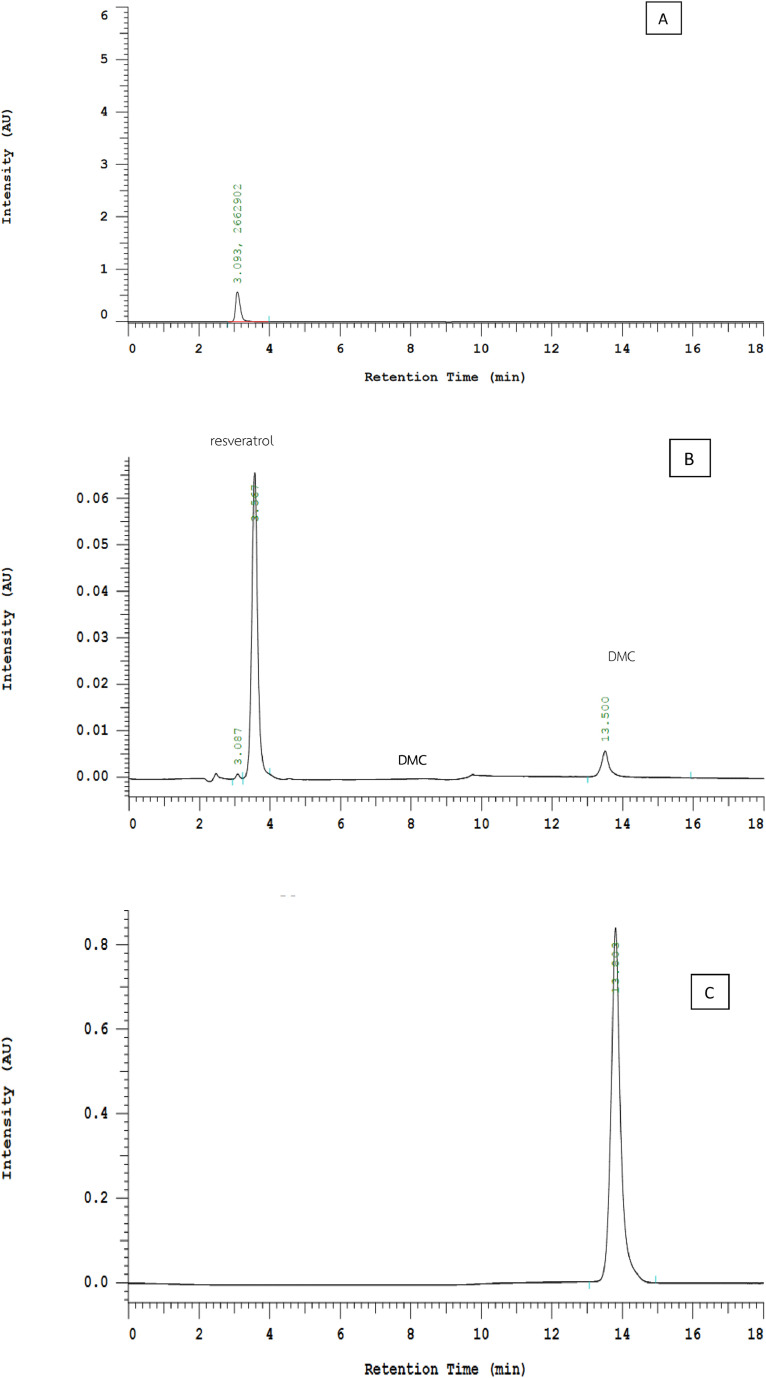
HPLC chromatograms blank pluronic nanomicelles [A]; mixture of DMC (10 mg/ml and resveratrol (10 mg/ml) under UV detector at 306 nm [B] and under fluorescence detector (excitation wavelength 420 nm; emission wavelength 470 nm) [C].

A reverse phase HPLC chromatographic system comprised of C-18 stationary phase (5 mm, 250 mm x 4.6 mm) for simultaneous quantitative determination of DMC and Res in Pluronic-F127 nanomicelle samples was utilized in the validation process. The HPLC analysis was carried out using a Hitachi CM-5000 equipped with a Hitachi Chromaster 5430 diode array detector and a Chromaster 5440 fluorescence detector with an autosampler. The optimum condition was a gradient system of mobile phase A (a mixture of 1 % MeOH + 0.1 % H_3_PO_4,_ pH 2.0 ± 0.1) and acetonitrile as mobile phase B. The% ratios of mobile phase A/mobile phase B were 55/45 (0–7 min), 30/70 (7.01–16 min), and 55/45 (16.01–18 min). The flow rate was maintained at 1 mL/min, and the column compartment was set at 40 °C with 5 µl injection volume. A UV detector at 306 nm was used for the detection of Res, and a fluorescence detector at excitation wavelength 420 nm and emission wavelength 470 nm was used for the detection of DMC. Our preliminary study using an isocratic system of 45 % acetonitrile and 55 % of a mixture of 1 % MeOH + 0.1 % H_3_PO_4,_ pH 2.0 ± 0.1 found that DMC under a fluorescence detector has a retention time of 47.4 min (Fig. 5). In contrast to using the above-mentioned gradient system, DMC reduced the retention time (about 3.5 folds) to 13.5 min (Fig. 6C), resulting in savings in assay time and solvents. Therefore, the gradient elution system was selected for method validation study.

The results demonstrated that the HPLC gradient system was specific for the assay and could be used selectively for the quantification of DMC and Res from pluronic nanomicelle samples. This can be seen from the chromatogram of the sample from blank nanomicelles (Fig. 6A), where no peak was observed at the same retention time for Res (about 3 min) and DMC (about 13 min) both under the UV detector (Fig. 6B) and fluorescence detector (Fig. 6C).

Moreover, the gradient HPLC elution system was sensitive, reproducible, and accurate for quantification of the 2 compounds: DMC and Res, in a single injection. The retention time of Res and DMC were 3.06 and 13.50 min, respectively. All peaks were well separated, with a resolution greater than 2.0. It is worth noting that Res could not be detected under a fluorescence detector. Therefore, the Res quantitative analysis was performed using the response from the UV detector, whereas DMC was determined using the fluorescence detector. The rationale behind the simultaneous use of two detectors was the sensitivity of the compounds towards the compounds. For instance, the low concentration of DMC in the formulation was unable to display a significant response in the UV detector. The optimized concentration of DMC used for preparation of the nano-micelles is very low, therefore the florescence detector was applied to quantify the DMC with low detection and quantification values. The time of flow of the mobile phase passing through the UV detector to the florescence detector was observed in seconds, which is considerably a shorter time making the simultaneous identification of the compounds in less time. Overall, the less time required, high precision and accuracy, convenience (identification through a single injection) and less solvent consumption are strengths of the method developed in our study.

### Linearity and range

The calibration curves of both DMC and Resveratrol were evaluated and covered 80 −120 % (2 - 25 µg/mL) of the assay concentration (10 mg/mL). It was found that the plots of peak area response from detectors against the concentrations of DMC and Res were linear [*Y* = 514733X + 30,449 and *Y* = 46873X - 4451.8 for DMC and Resveratrol, respectively, which was confirmed by the regression result of R^2^ value of 1.0 from both curves. Therefore, this range of both standard solutions will be used with confidence to evaluate the concentrations of both compounds in the nanomicelle samples.

### Limit of detection (LOD) and limit of quantitation (LOQ)

Since the developed method will be used for the determination of the releasing profiles of the encapsulated compounds from the nanomicelles after incubation in the selected releasing mediums, the effective analytical method should therefore be sensitive enough to be quantitively determined at low levels of concentration. The values of LOD and LOQ of Res and DMC are summarized in Tables 2 and 3. The results demonstrated that the LODs of Res and DMC were 0.164 and 0.371 µg/mL respectively.

**Table 2:** LOD and LOQ (µg/ml) of the analytes.

Standard	LOD (µg/ml)	LOQ (µg/ml)
DMC	0.371	1.237
Resveratrol	0.164	0.546

**Table 3:** Linear ranges and correlation coefficients of the calibration curves.

Standard	*Y* = aX + b linear model	r2	Concentration (µg/ml)
DMC	*Y* = 514733X + 30,449	1.000	2 - 25 µg/ml
resveratrol	*Y* = 46873X - 4451.8	1.000	2 - 25 µg/ml

*Y* = peak area, *X* = concentration (µg/ml).

### Accuracy

The mean percentage recovery (mean ± SD of the 2 standards in the mobile phase were calculated and reported in Table 4. The results of the accuracy study showed that all the readings from the samples were in the range of 100.09 to 100.76 %. The results demonstrated that the developed method gave an excellent accuracy value. Therefore, the method was suitable to determine the amount of each compound from the chemical stability and release studies.

**Table 4:** Recovery (%) of DMC in working standard solution.

QC sample (µg/ml)	Recovery (%) on each QC sample			Recovery (%) mean ±SD
	1	2	3	
8	101.00	101.73	102.47	101.73 ± 0.74
10	99.92	101.38	101.01	100.77 ± 0.76
12	99.35	99.38	98.80	99.18 ± 0.33
average	100.09	100.83	100.76	100.56 ± 0.41

## Repeatability

The repeatability of the method was determined by between-run analysis and within-run analysis. The repeatability of the method was expressed as% relative standard deviation (%RSD) of series of measurement. The experimental values obtained in the determination of the 2 compounds in standard solutions are presented in Table 5. The%RSD values demonstrated in Table 5 were lower than 2 %, which showed high repeatability for the method. The HPLC method validation results showed that the determination of DMC and resveratrol in QC samples could be performed by the validated HPLC method described above with accepted accuracy and precision. Table 6

**Table 5:** Recovery (%) of Resveratrol in working standard solution (QC sample).

QC sample (ug/ml)	Recovery (%) on each QC sample			Recovery (%) mean ±SD
	1	2	3	
8	100.78	100.58	100.73	100.70 ± 0.11
10	100.87	101.82	101.77	101.49 ± 0.53
12	101.26	101.32	101.45	101.34 ± 0.10
average	100.97	101.24	101.32	101.18 ± 0.18

**Table 6:** RSD (%) of between-run analysis and within-run analysis of DMC and resveratrol.

Concentration ``(µg/ml)	RSD (%)			
	DMC		Resveratrol	
	Between-run	Within-run	Between-run	Within-run
2	0.566	0.318	1.651	0.660
4	1.422	0.073	1.368	0.906
8	0.863	0.094	1.100	0.917
10	0.344	0.211	0.476	0.557
15	1.473	0.444	1.204	0.702
25	1.385	0.112	0.458	0.159

## Cytotoxicity evaluation

The cytotoxicity evaluation of DMC, resveratrol, their micelles and co-loaded micelles was carried out against MCF-7 and MDA-MB231 cell lines. The findings revealed that DMC alone showed higher toxicity against both MCF-7 and MDA MB231 cells, with IC_50_ = 18.21 ± 1.98 and 36.45 ± 1.22 µM, respectively while its micelles presented lower toxicity with IC_50_ = 22.35 and 50.55 µM, respectively (Table 7). But their co-loading in micelles presented an enhance cytotoxicity against both cell line with IC_50_ values of IC_50_ = 15.22 and 22.21 µM, respectively. From the results it is evident that the co-loading of DMC and Res enhanced the anticancer activity of DMC.

**Table 7:** The IC_50_ values of DMC, resveratrol and doxorubicin against MCF-7 and MDA-MB 231.

Sample	MCF-7	MDA-MB 231
	IC_50_ (µM)	IC_50_ (µM)
DMC	18.21±1.98	36.45±1.22
Resveratrol	169.22±6.47	325.78±7.23
DMC-micelles	22.35±1.12	50.55±2.01
Res-micelles	209.22±3.78	330.21±4.13
DMC-Res micelles (IC_50_ of DMC)	15.22±0.97	22.21±0.89
DMC-Res micelles (IC_50_ of Res)	301.12±4.34	321.17±4.98

All the values are expressed as mean ± standard deviation (*n* = 3). DMC: dimethylcurcumin; Res: Resveratrol.

Overall, this study presented a convenient, precise and accurate HPLC method was developed and validated for simultaneous quantitative determination of co-loaded drugs dimethylcurcumin (DMC) and resveratrol (Res) in polymeric nano-micelles through a single injection with a shorter retention time. Moreover, an advance approach was used for the enhancement of the anticancer efficacy of dimethylcurcumin (DMC) by combining it with resveratrol (Res) and loading it into polymeric nanomicelles. The combination of the drugs alone did not show significant activity against MCF-7 and MDA-MB 231, while the dimethylcurcumin showed higher efficacy against the cancer cell lines compared to standard DMC. The findings strongly encourage the application of the method for the quantitative assay and the combined loading of DMC and Resveratrol in polymeric nanomicelles for enhanced anticancer activity. This study also warrants some further investigations on the toxicity profile of the formulated system.

## Ethics statements

Not applicable

## CRediT authorship contribution statement

**Wasiporn Arakkunakorn:** Methodology, Investigation, Validation, Writing – original draft. **Watchara Pholthien:** Investigation, Validation. **Warayuth Sajomsang:** Methodology, Supervision. **Abdul Basit:** Methodology, Writing – review & editing. **Sasikarn Sripetthong:** Methodology, Investigation, Validation, Writing – original draft. **Sirinporn Nalinbenjapun:** Methodology, Investigation, Validation, Writing – original draft. **Chitchamai Ovatlarnporn:** Conceptualization, Supervision, Resources, Validation, Writing – review & editing.

## Declaration of Competing Interest

The authors declare that they have no known competing financial interests or personal relationships that could have appeared to influence the work reported in this paper.

## Data Availability

No data was used for the research described in the article.
